# Morbid Matter in the Blood

**Published:** 1854-04

**Authors:** 


Morbid Matter in the Blood.—Mr. Henry Lee exhibited several preparations illustrative of the influence of vitiated secretions in the blood in the production of fibrinous deposits.

Dr. Routh remarked, that experiments with reference to the influence of the injection of vitiated secretions into the circulation had been attended with very contradictory results. In some cases the symptoms and effects produced were similar to those mentioned by Mr. Lee; in other instances no perceptible effect was produced. So in cases of dissection-wound some persons suffered, some did not. This he thought showed that the effects were not attributable merely to the introduction of the morbid matter, but that other causes were in action, such perhaps as some epidemic influence. The persons affected also were in a low state of health. As to the statement that the presence of pus in the blood always produced hectic fever, he could not believe it, and thought that facts were opposed to such a statement.

Mr. Henry Lee remarked that the occurrence of fibrinous deposits in some of the cases experimented upon proved his case. Rabies was not always the result of innoculation of the poison, but no one presumed therefore to deny the influence of the *morbus materia.*

Dr. Mackenzie had no hesitation in siding with Mr. Henry Lee on the question of the obstruction of veins by fibrinous deposition from a vitiated condition of the blood. He did not speak specifically of the vitiated condition of the blood from pus, because this, being a fluid which varied very greatly in its properties, would give rise to very different results in different cases. Speaking, however, of vitiation of the blood generally, he could affirm that from this cause alone, arrest, stagnation, and coagulation of blood might take place in the veins, without any primary inflammation of their coats. That some altered state of their lining membrane was induced by the vitiating matter he had no doubt, although it was difficult to define its exact nature, and he believed that in consequence of this alteration, the blood, instead of flowing onwards, was arrested, and coagulated in the veins, upon which arrest and coagulation the general phenomena of phlebitis ensued. —*Lancet.*

